# Fabrication of 3-methoxyphenol sensor based on Fe_3_O_4_ decorated carbon nanotube nanocomposites for environmental safety: Real sample analyses

**DOI:** 10.1371/journal.pone.0177817

**Published:** 2017-09-22

**Authors:** Mohammed M. Rahman, Mohammad Musarraf Hussain, Abdullah M. Asiri

**Affiliations:** 1 Chemistry Department, King Abdulaziz University, Jeddah, Saudi Arabia; 2 Center of Excellence for Advanced Material Research (CEAMR), King Abdulaziz University, Jeddah, Saudi Arabia; Institute of Materials Science, GERMANY

## Abstract

Iron oxide ornamented carbon nanotube nanocomposites (Fe_3_O_4_.CNT NCs) were prepared by a wet-chemical process in basic means. The optical, morphological, and structural characterizations of Fe_3_O_4_.CNT NCs were performed using FTIR, UV/Vis., FESEM, TEM; XEDS, XPS, and XRD respectively. Flat GCE had been fabricated with a thin-layer of NCs using a coating binding agent. It was performed for the chemical sensor development by a dependable I-V technique. Among all interfering analytes, 3-methoxyphenol (3-MP) was selective towards the fabricated sensor. Increased electrochemical performances for example elevated sensitivity, linear dynamic range (LDR) and continuing steadiness towards selective 3-MP had been observed with chemical sensor. The calibration graph found linear (*R*^2^ = 0.9340) in a wide range of 3-MP concentration (90.0 pM ~ 90.0 mM). The limit of detection and sensitivity were considered as 1.0 pM and 9×10^−4^ μAμM^-1^cm^-2^ respectively. The prepared of Fe_3_O_4_.CNT NCs by a wet-chemical progression is an interesting route for the development of hazardous phenolic sensor based on nanocomposite materials. It is also recommended that 3-MP sensor is exhibited a promising performances based on Fe_3_O_4_.CNT NCs by a facile *I-V* method for the significant applications of toxic chemicals for the safety of environmental and health-care fields.

## Introduction

The protection is a key apprehension in viewpoint of atmosphere and health that is a great issue to examine using sensors intended for the identification & recognition of toxic materials through an established practice. Semiconductor nanostructure material is very proficient and perceptive due to their high active surface area and different spherical morphologies to volume ratio in comparison with typical diameter from nano to micro ranges. In recent times, the nanostructure of metal oxide is an immense interest having their fascinating criteria such as fabrication of chemical sensor, dynamic surface area, elevated porosity, permeability, quantum confinement consequence, and stability [1–21]. Sensor based metal oxide conjugated carbon composites are extensively used for the monitoring of air-water contamination, chemical process and poisonous constituents in the environment [22, 23]. Recognition and partition of contaminated resources from industrial waste water is one of the key issues in the biological and environmental field. Different methods reported for the isolation and removing of carcinogenic materials from the industrial waste water but a few issues are still remaining troubled that are removing of toxic agents in efficiently and re-usability of the NCs materials including their preparation at a facile and low cost. The mesoporous character of the NCs material allows a simplistic recycling devoid of foremost degradation of sensor effectiveness and potentiality. An excellent absorption and adsorption ability of the hybrid NCs, makes it’s an appropriate sensor for the identification and removing of marked harmful agents from industrial and environmental wastes. Substituted and un-substituted phenols are common bi-products of industrial process having high toxicity properties. They are frequent contaminants in food, fresh and waste water [24]. 3-MP is an effective toxic element to environment and health. Hence it is very important to expand a suitable analytical process which is dependable, economical and efficient for the accurate quantification and sensitive finding of 3-MP. Various sensing techniques have been reported in the previous study to detect phenolic compounds such as electrochemical methods, HPLC and spectrometry. Among several detection methods, the electrochemical current—voltage (I-V*)* technique is a cheap, portable and easy to implement. Therefore, based on different nanostructure materials, semiconductor undoped or doped nanomaterials (NMs), transition metal oxides, electrocatalytic moieties, several chemically modified electrodes have been developed for the detection of 3-MP [25].

Diverse classes of nanoparticles (NPs), metallic or polymeric colloids used to improve the patient compliance and therapeutic efficiency of applicable medicines. Ferro-fluids are stable dispersions in water phase of magnetic iron-oxide NPs which have been studied in biomedical sciences as proficient device in *vitro* diagnosis, cell separation, immunoassays and nucleic acid concentration [26]. In chemically, iron oxide NPs have been used in NO reduction [27], adsorbents for heavy metals [28], pigments in cosmetic powders [29], anodes in lithium ion-batteries [30], detection of hydrogen peroxide [31], polymer coated of supra-magnetic NPs [32], application in magnetic resonance imaging [33], biomedical applications [34], imaging agents [35], photo-catalysis [36], removing of inorganic and organic pollutants [37], glycerol hydrogenolysis [38], hydrogenation of nitrobenzene [39], application in high-performance supercapasitor [40], catalytic oxidation [41], water treatment [42], separation of acid dye [43], antibody functionalization [44], biosensor applications [45], hybridization of nanotube [46], oil spill removing [47] and bio-distribution studies [48]. In this approach, Fe_3_O_4_.CNT NCs prepared by a simple wet-chemical process in alkaline phase, which revealed a steady growth development of NMs onto CNT surfaces and significantly executed for their potential applications. Fe_3_O_4_.CNT NCs have been used to fabricate a simple and efficient chemical sensor and assessed for the sensing performance selectively considering 3-MP in phosphate buffer (PB) at room temperature. To the best of our knowledge, this is the initial report for detection of 3-MP with prepared Fe_3_O_4_.CNT NCs onto GCE using an easy, suitable, and dependable I-V technique with short response time.

## Experimental section

### Materials and methods

The analytical grade chemicals such as acetone (Ac), 4-aminophenol (4-AP), ammonium hydroxide (NH_4_OH), carbon nanotube (CNT), disodium phosphate (Na_2_HPO_4_), ethanol (EtOH), ferrous sulfate (FeSO_4_.7H_2_O), hydrazine (Hy), 3-methoxyphenol (3-MP), 4-methoxyphenol (4-MP), monosodium phosphate (NaH_2_PO_4_), nafion (5% ethanolic solution), *n*-hexane (Hx), 2-nitrophenol (2-NP), sodium hydroxide (NaOH), tetrahydrofuran (THF), tolune-4-sulfonic acid hydrazide and xanthine (Xn), purchased from Sigma-Aldrich Company and used as received. FT-IR and UV/V spectra of the dried brown Fe_3_O_4_ NPs, and Fe_3_O_4_.CNT NCs were performed using Thermo scientific NICOLET iS50 FTIR spectrometer (Madision, WI, USA) and 300 UV/Visible spectrophotometer (Thermo scientific) respectively. The XPS measurements were examined to calculate the binding energy (eV) of C, Fe and O on a K-α spectrometer (Thermo scientific, K-α 1066) with an excitation radiation source (A1 Kα, Beam spot size = 300.0 μm; pass energy = 200.0 eV; pressure ~ 10^−8^ torr). The morphology and particle size of CNT, Fe_3_O_4_ NPs, and Fe_3_O_4_.CNT NCs were analyzed by FESEM and TEM (JEOL, JSM-7600F, Japan). XRD experiment was also carried out under ambient conditions to detect the crystalline pattern of Fe_3_O_4_.CNT NCs. *I-V* was performed [49, 50] to select 3-MP at a specific point by fabricated Fe_3_O_4_.CNT NCs using Keithley electrometer (6517A, USA) under room conditions.

### Growth mechanism of Fe_3_O_4_.CNT NCs

**Fig 1:**
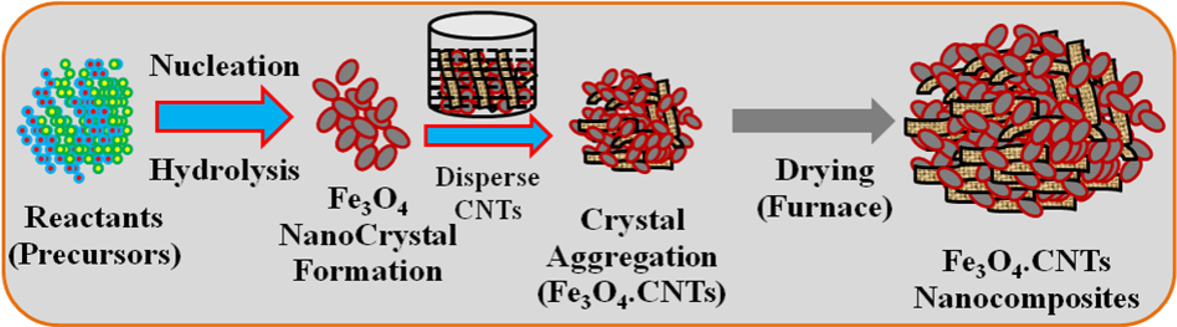
Schematic representation of growth mechanism of Fe_3_O_4_.CNTs NCs by a wet-chemical process.

Preparation of the Fe_3_O_4_.CNT nanocomposites is explained in detail and presented in the supporting information section (S1 Fig). In Fe_3_O_4_.CNT NCs growth method, initially Fe_3_O_4_ nucleus growth takes place by itself & mutual-aggregation, then nano-crystal re-aggregated and formed aggregated Fe_3_O_4_ nanocrystal using Ostwald-ripening method. Nanocrystal crystallizes and re-aggregates with each counter part in presence of disperse CNT through Vander-Waals forces and reformed Fe_3_O_4_ decorated CNT onto porous carbon nanotubes morphology, which presented in **Fig 1.**

### Fabrication of glassy carbon electrode with Fe_3_O_4_.CNT NCs

NaH_2_PO_4_ (0.2 M, 39.0 mL), Na_2_HPO_4_ (0.2 M, 61.0 mL), and distilled water (100.0 mL) had been used for the preparation of PB (200.0 mL, 100.0 mM, pH = 7). Ethanol and conducting binder, nafion were used to fabricate GCE (surface area = 0.0316 cm^2^) with Fe_3_O_4_.CNT NCs. After that, the fabricated electrode was kept at R. T. (3 h) for uniform film formation with completed drying. The fabricated GCE and platinum (Pt) were used as a working and counter electrode respectively in order to find out the I-V signals.

## Results and discussion

### Evaluation of optical and structural properties

**Fig 2:**
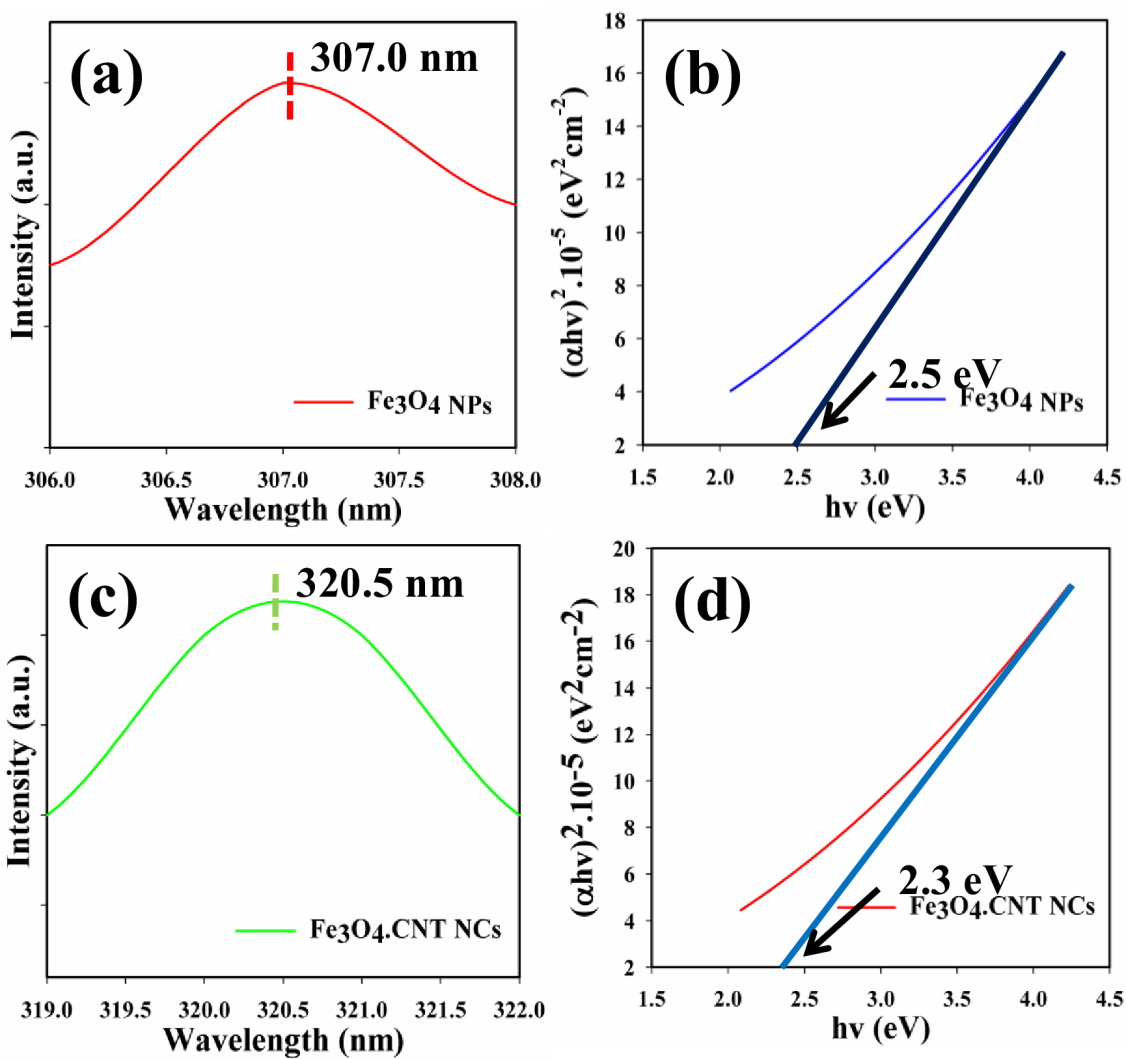
(a-c) UV/Vis spectra and (b-d) Band-gap energy plot of Fe_3_O_4_ NPs and Fe_3_O_4_.CNT NCs.

The optical property is one of the important characteristics for the assessment of photo-catalytic activity of the brown grown Fe_3_O_4_ NPs and Fe_3_O_4_.CNT NCs. Based on UV/Vis. theory, the outer electrons of the atom absorb radiant energy and then shifted to the higher-energy levels. The spectrum including band-gap energy of the metal oxide can be achieved due to the optical absorption. The UV/Vis. spectra of the Fe_3_O_4_ NPs and Fe_3_O_4_.CNT NCs were recorded in the visible range (200 ~ 800 nm). The absorption band at around 307.0 and 320.5 nm were found respectively (Fig 2A–2C). Based on the maximum level of band absorption, the band-gap energies of the Fe_3_O_4_ NPs and Fe_3_O_4_.CNT NCs were calculated using Tauc’s equation (vi). Here, α = Absorption coefficient, A = Constant related to the effective mass of the electrons, r = 0.5 (Direct transition), E_g_ = Band-gap energy, h = Plank’s constant, v = Frequency. Following the direct band-gap rule (*αhv*)^2^ = *A* (*hv-E*_*g*_), curve of (*αhv*)^2^ *vs hv* was plotted and then extrapolated to the axis. From the extrapolated curve, the band-gap energies for Fe_3_O_4_ NPs and Fe_3_O_4_.CNT NCs were found as 2.5 and 2.3 eV correspondingly (Fig 1B–1D) [51–53].

$${\left(\alpha hv\right)}^{1/r}=A\left(hv\text{-}E_{g}\right)$$ (vi)

**Fig 3:**
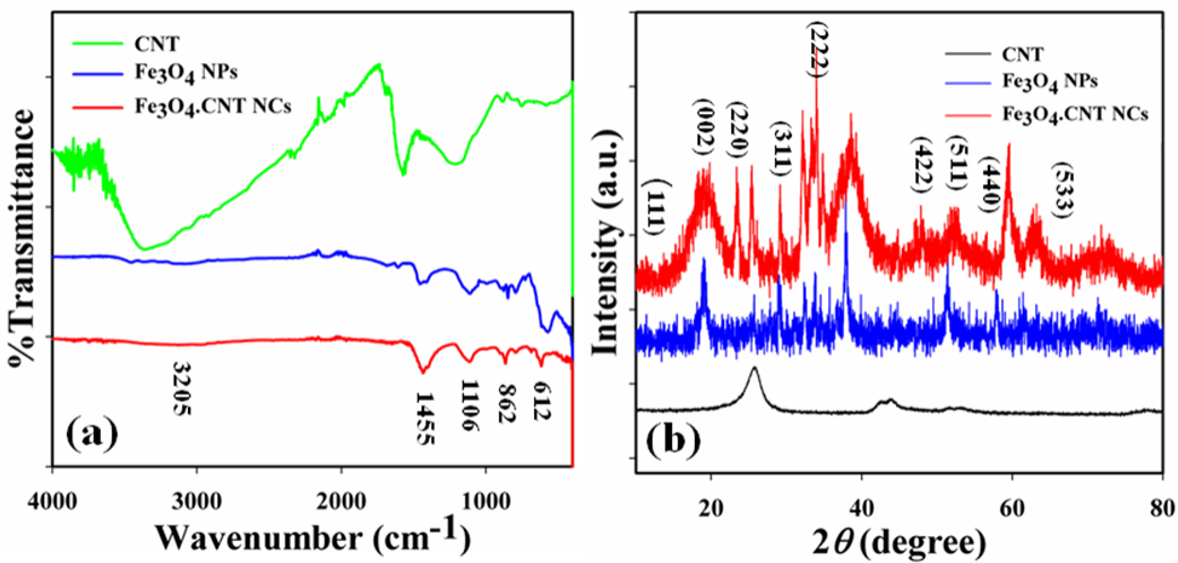
(a) FT-IR spectra, and (b) XRD patterns of CNT, Fe_3_O_4_ NPs and Fe_3_O_4_.CNT NCs.

The CNT, Fe_3_O_4_ NPs and Fe_3_O_4_.CNTs NCs were also examined in perception of atomic and molecular vibrations to recognize the functional nature of the NCs using FTIR, and spectra were recorded in the region of 4000~400 cm^-1^ under room conditions. The FTIR spectra of the NCs shows peaks at 3205 (br), 1455 (s), 1106 (m), 862 (m) and 612 (m) cm^-1^ which recognized the presence of O-H (stretching), C−H (rocking), Fe−O−Fe (stretching), C−H and Fe = O (stretching) respectively (**Fig 3A**). The peak at 612 cm^-1^ indicates the formation of metal-oxide (Fe-O) bond which recognized the configuration of the Fe_3_O_4_ NPs and Fe_3_O_4_.CNT NCs [54].

Generally, the crystalline pattern indicates the metal-oxygen framework in nanostructure materials. XRD analysis was conducted to observe the crystalline nature of prepared Fe_3_O_4_.CNT NCs. The potential peaks with indication for 2*θ* values at 18.0 (111), 24.0, 25.5 (002), 29 (220), 32.0, 34.0 (311), 38.0 (222), 52.0 (422), 59.5 (511), 64.0 (440) and 73.0 (533) degrees (**Fig 3B**) were observed. All the pragmatic peaks in the spectra were assigned by using the JCPDS file (019–0629). The observed peak at 25.5 (002) was denoted for carbon of CNT and NCs. The strongest peak indicates the crystalline pattern and purity of the NCs. From the XRD analysis, it was suggested that a big amount of crystalline Fe_3_O_4_ was present in the synthesized iron oxide decorated CNT NCs [55].

### Morphological and elemental characteristics

**Fig 4:**
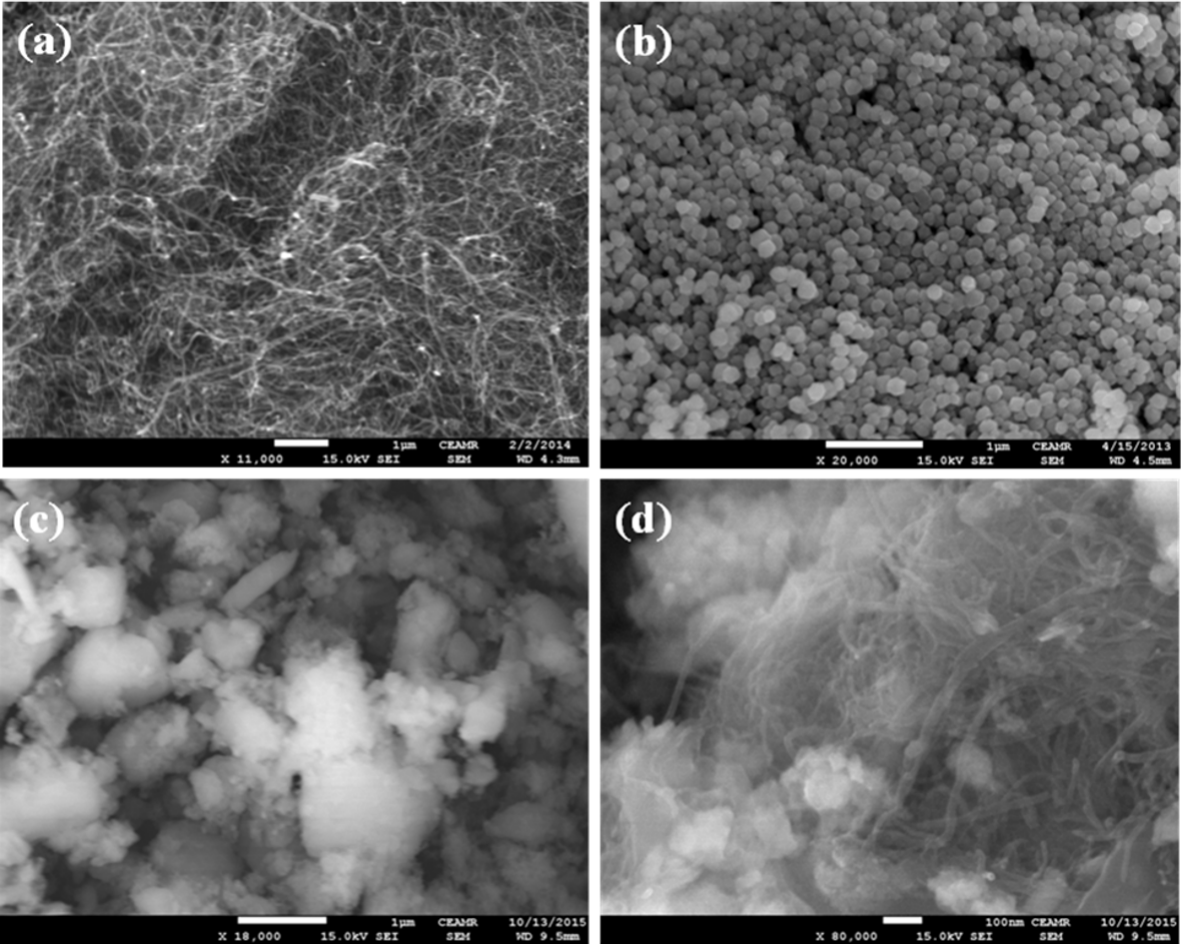
Magnified FESEM images (a) CNT, (b) Fe_3_O_4_ NPs, and (c-d) Fe_3_O_4_.CNT NCs.

FESEM is one of the well-recognized processes to observe the morphology of the materials. The morphology and elemental analysis of the prepared brown Fe_3_O_4_.CNT NCs were measured using FESEM coupled-XEDS respectively. The typical shapes of CNT, Fe_3_O_4_ NPs, and brown Fe_3_O_4_.CNT NCs had been recorded from low to high magnified images (**Fig 4A–4D**). According to the magnified images, Fe_3_O_4_ was aggregated and decorated with a bright contrast along with well-dispersed onto the CNT surfaces. The conductance of CNT may be increased with the addition of Fe_3_O_4_ which correlated the calculation of band-gap energy (*E*_bg_) of two different molecules.

**Fig 5:**
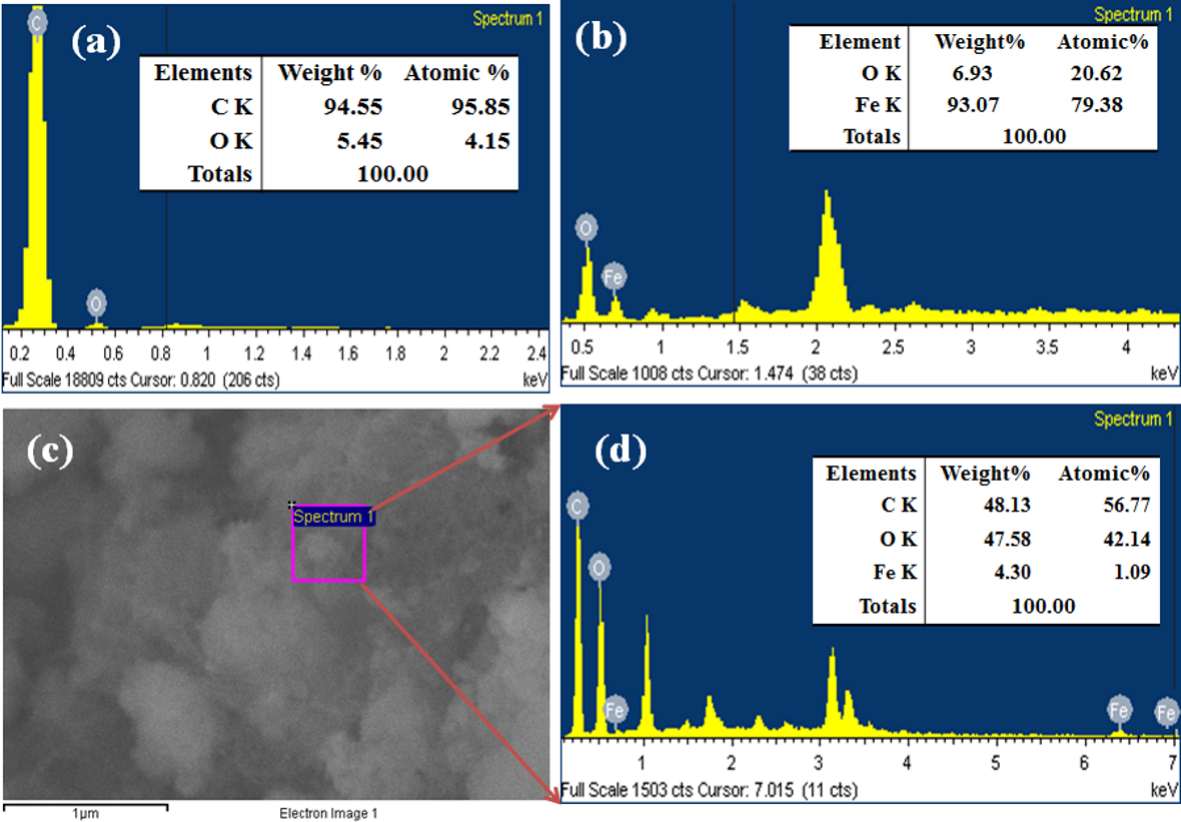
Elemental analysis (a) CNT, (b) Fe_3_O_4_ NPs, and (c-d) Fe_3_O_4_.CNT NCs.

Upon analysis of XEDS, oxygen (O) and iron (Fe) & carbon (C), oxygen (O) and iron (Fe) were found in the synthesized brown Fe_3_O_4_ NPs and Fe_3_O_4_.CNT NCs and contains O (6.93), Fe (93.07) & C (48.13), O (47.58) and Fe (4.30) wt% respectively. On the basis of FESEM equipped XEDS spectra, C are present in NCs but absent in NPs. There are no other peaks related with impurities were found in the spectra which indicated that the NCs are composed of C, O, and Fe only (**Fig 5A–5D**).

### Determination of binding energy

**Fig 6:**
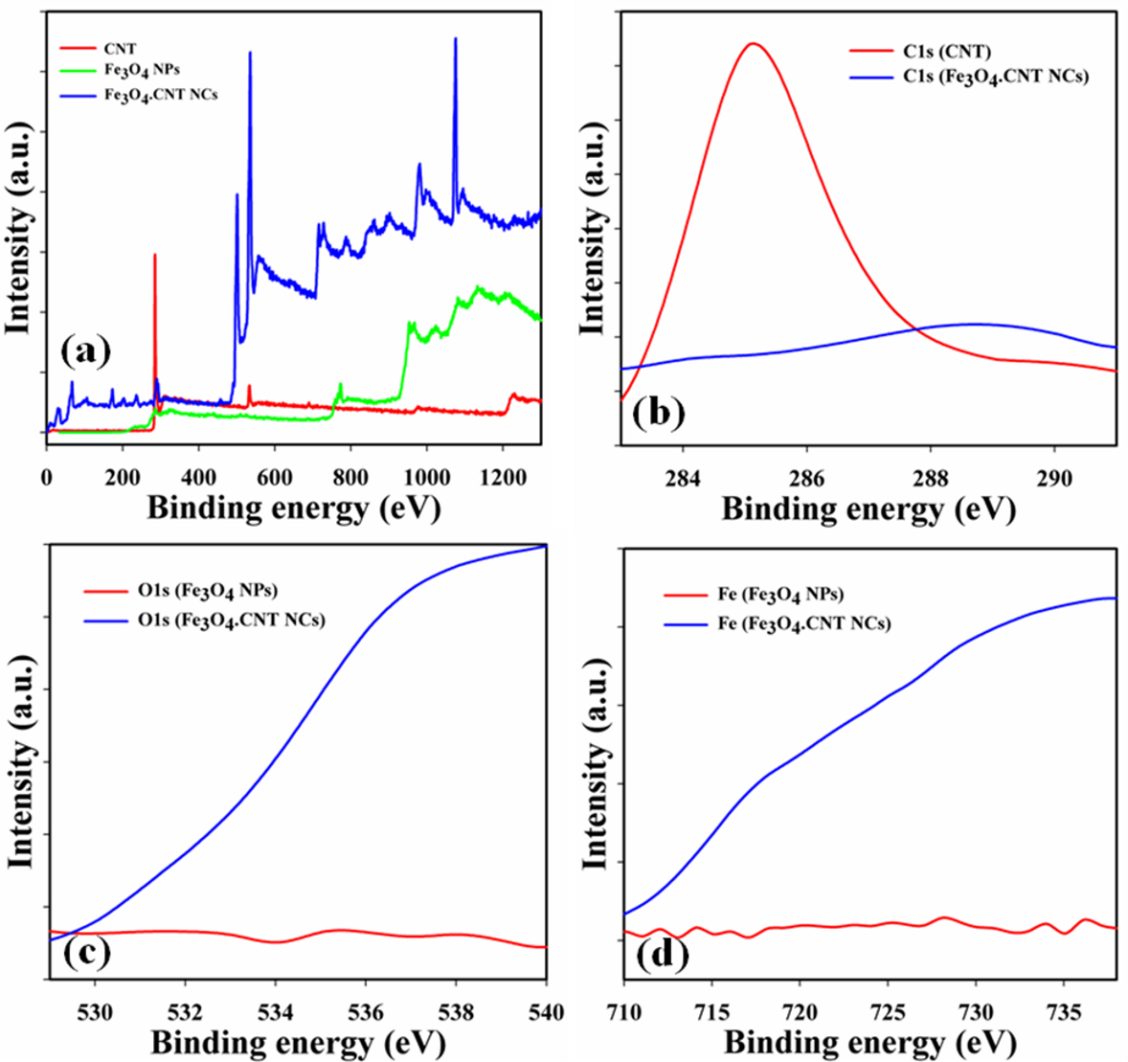
XPS study of CNT, Fe_3_O_4_ NPs, and Fe_3_O_4_.CNT NCs (a) Full spectrum, (b) C1s level, (c) O1s, and (d) Fe^2+^ 2p_3/2_ and Fe^2+^ 2p_1/2_ level.

XPS is a quantitative spectroscopic system which can be used to indicate the chemical nature of the elements present in the NCs. XPS spectra may be recorded by irradiating of an X-ray beam with a NCs material and kinetic energy including electrons number of the sample can be determined consecutively. According to the XPS spectra, carbon, oxygen and iron were found in the prepared Fe_3_O_4_.CNT NCs. A comparison between binding energies among CNT, Fe_3_O_4_ NPs and Fe_3_O_4_.CNT NCs are presented in **Table 1**and Fig 6A–6D [56].

**Table 1:** Binding energies of NMs.

Elements	C1s	O1s	Fe^2+^ 2p_3/2_	Fe^3+^ 2p_3/2_	Fe^2+^ 2p_1/2_	Fe^3+^ 2p_1/2_
CNT	285.0	-	-	-	-	-
Fe_3_O_4_ NPs	-	553.0	712.0	717.0	728.0	734.0
Fe_3_O_4_.CNT NCs	289.7	535.4	710.3	716.4	721.2	725.1

### TEM analysis

**Fig 7:**
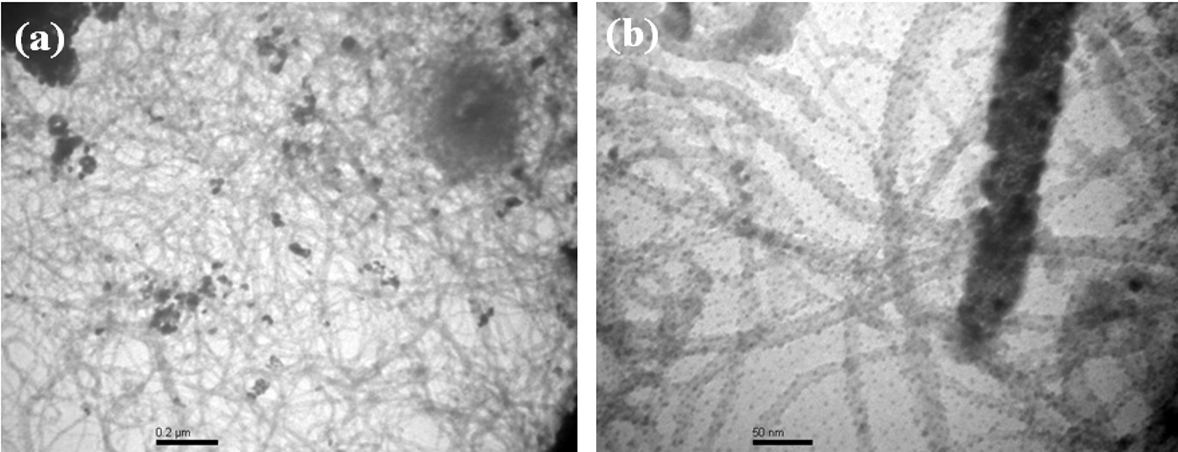
TEM analysis of Fe_3_O_4_.CNT nanocomposites (a-b) Low-to-high magnified images.

Additional morphological evaluation of Fe_3_O_4_.CNT nanocomposites was investigated by TEM analysis. It is revealed that the aggregated spherical-shaped Fe_3_O_4_ nanoparticle decorated onto CNT morphology, which is presented in Fig 7A and 7B. The TEM images (Fig 7A and 7B of Fe_3_O_4_ NPs decorated nanocomposites of CNT were showed the existence of aggregated Fe_3_O_4_ nanoparticle adsorption onto the surface of CNTs nanocomposites. In the TEM images, it displays the actual morphology of the various nanocomposites assembled in spherical-shaped Fe_3_O_4_-particle-like morphology decorated CNT, which correspondence to the adsorption as well as aggregation of nanocomposite materials.

## Application

### Detection of 3-methoxyphenol by Fe_3_O_4_.CNT NCs

**Fig 8:**
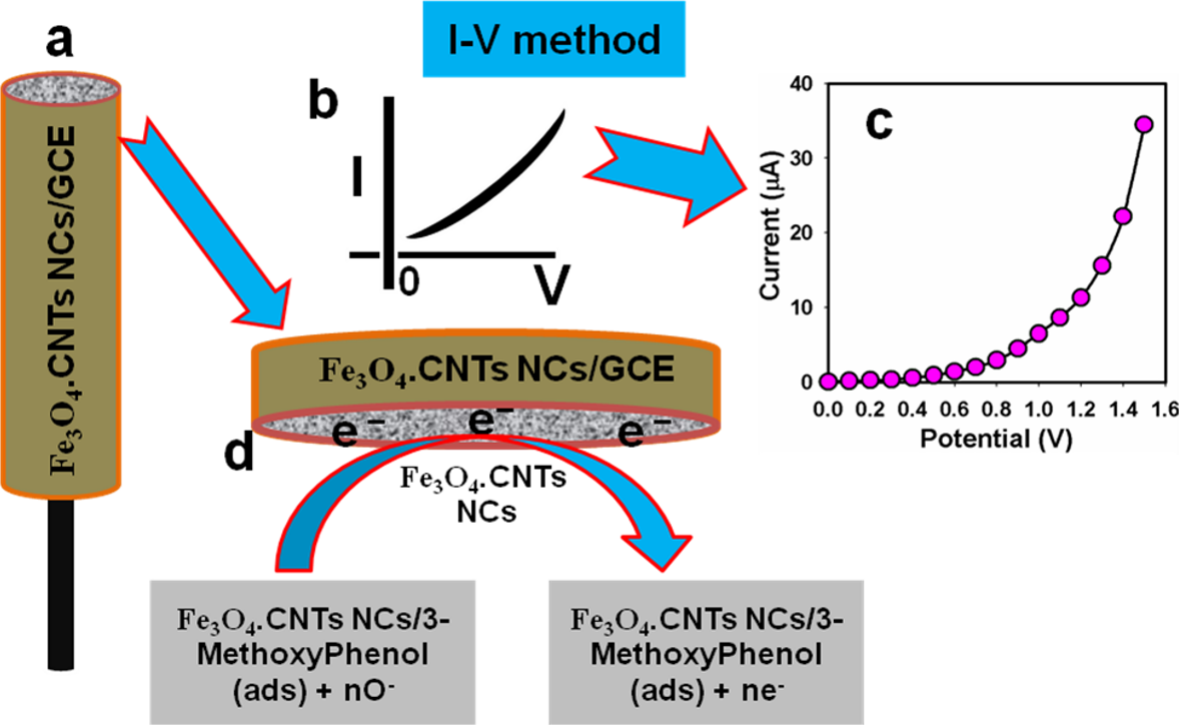
Schematic view (a) Coated rod-shape round disc-GCE, (b) Expected *I-V* curve, (c) Observed *I-V* response, (d) Proposed detection mechanism of 3-MP by Fe_3_O_4_.CNT NCs/GCE.

Enhancement of the fabricated electrode with NCs is the initial stage of using as a chemical sensor. The significant application of Fe_3_O_4_.CNT NCs is assembled onto GCE as a chemical sensor, which carried out for the detecting and measuring of target agent, 3-MP in PB. The Fe_3_O_4_.CNT NCs/GCE sensor have more advantages for example chemically inert, safe, electro-chemical activity, easy to fabricate, non-toxic, simple to assemble and stable in air. According to the I-V method, the current responses of Fe_3_O_4_.CNT NCs/GCE were considerably changed during 3-MP adsorption. A significant amplification in the current response with applied potential was noticeably confirmed having the holding time of electrometer was 1.0 sec. The overall possible mechanism of 3-MP detection by Fe_3_O_4_.CNT NCs using I-V technique is presented in **Fig 8**.

**Fig 9:**
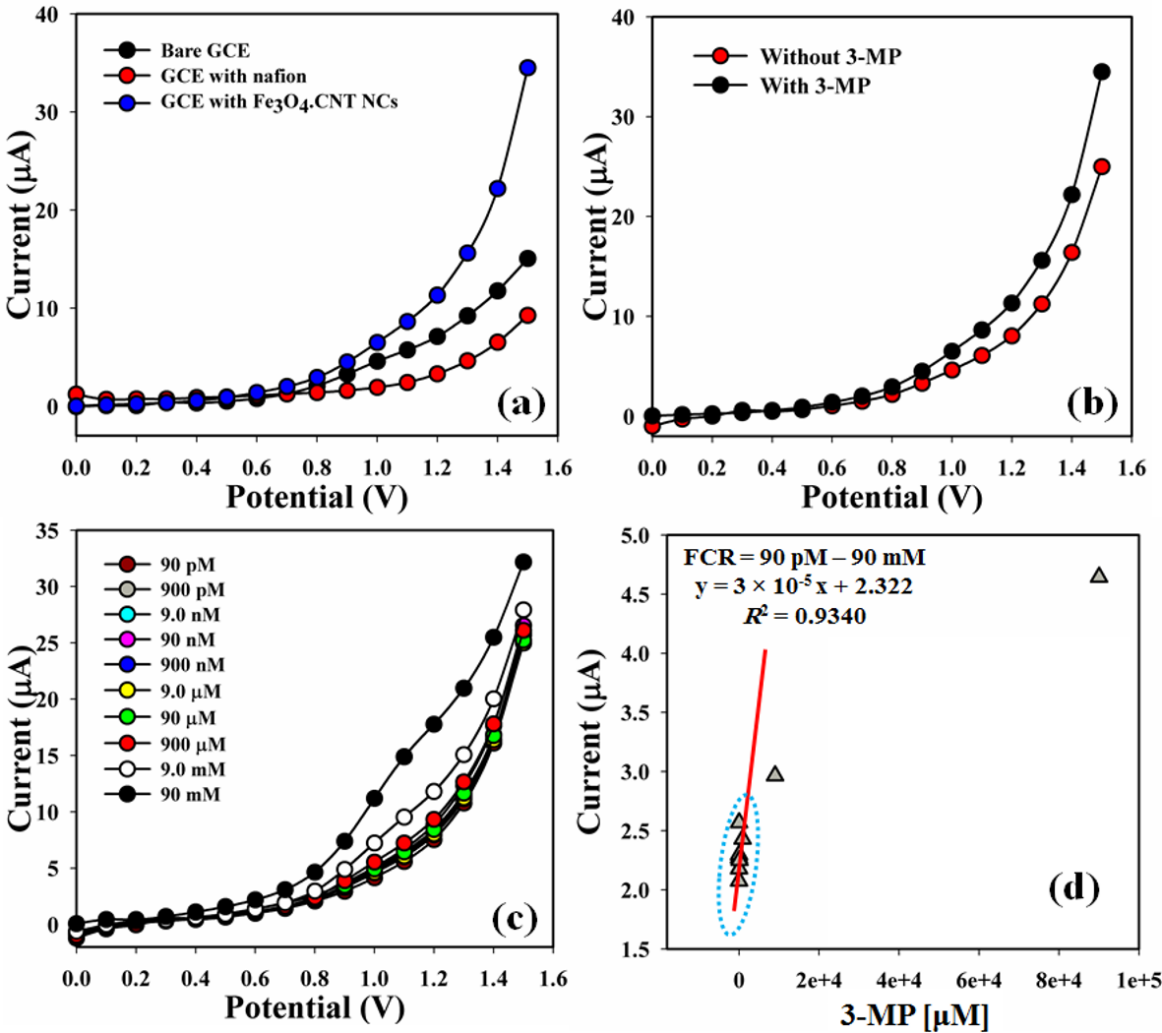
Current-voltage responses of Fe_3_O_4_.CNT NCs (a) Bare and coated electrode, (b) Absence and presence of 3-MP, (c) Concentration variation of the 3-MP and (d) Calibration curve.

**Fig 10:**
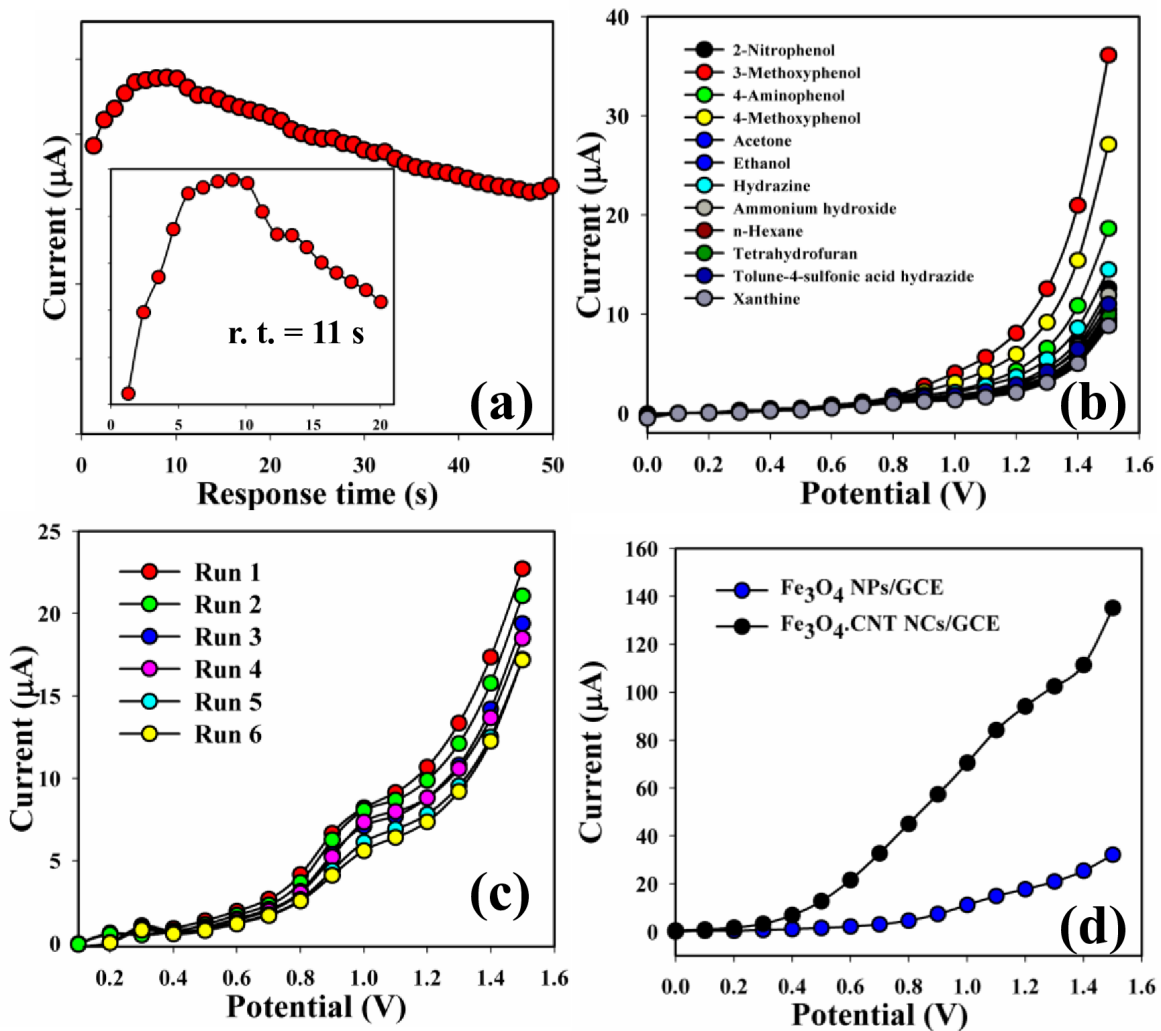
(a) Response time, (b) Selectivity, (c) Reproducibility study, and (d) Control experiment.

The potential application of Fe_3_O_4_.CNT NCs assembled onto an electrode as a chemical sensor has been engaged for the identification of compounds that are biological and environmentally hazardous. The current responses (potential range: 0 ~ +1.5 V) for the bare, GCE with nafion, and coated with Fe_3_O_4_.CNT NCs on the working electrode surface were presented in **Fig 9A.** The differences of the current responses between bare and coated GCE occurred due to the current signals were enhanced by coated electrode in compared with bare GCE. The current signal without (red-dotted) and with (black dotted) analyte were recorded (**Fig 9B**). A significant improvement of current responses occurred in case of the modified Fe_3_O_4_.CNT electrode with 3-MP which gives a higher surface area with better coverage in absorption and adsorption potentiality onto the porous NCs surfaces of the target compound (3-MP). The I-V responses of the 3-MP with different concentration (90 pM ~ 90 mM) towards Fe_3_O_4_.CNT NCs modified electrode were recorded which signified that the changes of current of the fabricated electrode was a function of 3-MP concentration under normal condition and it was also revealed that the current responses increased regularly from lower to higher concentration of the target molecule (**Fig 9C**). A broad range of the analyte concentrations were measured from the lower to higher potential (0.0 ~ 1.5 V) to examination of the possible analytical limit. The linear calibration curve at 0.8 V were plotted from the various concentrations of 3-MP (90 pM ~ 90 mM). The LDR (90 pM ~ 90 nM), regression co-efficient (*R*^*2*^ = 0.9340), sensitivity (9 × 10^−4^ μAμM^-1^cm^-2^), and LOD (1.0 pM) at signal to noise ratio ~ 3 were calculated from the calibration curve (**Fig 9D**). Response time (r. t. = 11 s) of the electrode was calculated from the practical concentration variation graph (**Fig 10A**).

The resistance value of the Fe_3_O_4_.CNT NCs modified GCE chemical sensor can be decreased with increasing active surface area which is an important property of the growth NCs particles [57]. These reactions could be occurred in bulk-system/air-liquid interface/neighboring atmosphere owing to the small carrier concentration, which increased the resistance during increasing the electrical properties. For enhancement of the oxygen adsorption, the sensitivity/conductivity of 3-MP towards Fe_3_O_4_.CNT NCs could be ascribed having higher-oxygen lacking conducts. Larger amount of oxygen adsorbed on the Fe_3_O_4_-doped NCs sensor surface, higher would be the oxidizing potentiality and faster would be the oxidation of 3-MP and higher would be the resultant current. The activity of 3-MP would have been extremely big as contrast to other toxic chemical with the surface under indistinguishable conditions [58, 59]. In two-electrode system, I-V characteristic of the Fe_3_O_4_.CNT NCs coated GCE is activated as a function of 3-MP concentration at room conditions, where improved current response was observed. As obtained, the current response of the Fe_3_O_4_.CNT NCs/GCE film was increased with the increasing concentration of 3-MP; however similar phenomena for toxic chemical detection have also been reported earlier [60–62]. At a low concentration of 3-MP in liquid medium, there is a smaller surface coverage of 3-MP molecules on Fe_3_O_4_.CNT NCs/GCE film and hence the surface reaction proceeds steadily. By increasing the 3-MP concentration, the surface reaction is increased significantly (gradually increased the response as well) owing to large surface area contacted with 3-MP molecules. Further increasing of 3-MP concentration on Fe_3_O_4_.CNT NCs/GCE surface, it was exhibited a more rapid increased of current responses, due to larger surface covered by 3-MP. The 3-MP sensing mechanism of the Fe_3_O_4_.CNT NCs/GCE fabricated film is explained and presented in reactions [vii—ix]. Where, oxygen (dissolved) is chemisorbed on the Fe_3_O_4_.CNT NCs/GCE surfaces, when the porous-fabricated-film is immersed in PB. During the chemical adsorption, the dissolved oxygen is transferred into ionic species such as O_2_^−^ and O^−^ which gained electrons from the conduction band.

$$O_{2(diss)}\to O_{2(ads)}$$ (vii)

$$O_{2(ads)}+e^{-}\to O_{2}{{}^{-}}_{\left(ads\right)}$$ (viii)

$$O_{2}{{}^{-}}_{\left(ads\right)}+e^{-}\to 2O^{-}{}_{\left(ads\right)}$$ (ix)

The reaction between 3-MP and ionic oxygen species can take place in (x), and the reaction is depended on the concentration of 3-MP in the medium. On Fe_3_O_4_.CNT NCs/GCE surfaces, 3-MP oxidized and then electrons were released into the conduction band, therefore decreased the resistance and consequently increased the transmission current.

$$3\text{-}MP\;\left(ad\text{-}ox/Fe_{3}O_{4}.CNT\right)+nO^{-}{}_{\left(ads\right)}\to 3\text{-}MP\;\left(de\text{-}red/Fe_{3}O_{4}.CNT\right)+ne^{-}$$ (x)

Response time was measured by Fe_3_O_4_.CNT NCs/GCE in presence target 3-MP analyte and presented in **Fig 10A**. The selectivity was performed with different chemicals such as 2-NP, 3-MP, 4-AP, 4-MP, Ac, EtOH, Hy, NH_4_OH, Hx, THF, tolune-4-sulfonic acid, hydrazide, and Xn. 3-MP showed maximum current responses towards Fe_3_O_4_.CNT NCs fabricated electrode and therefore it was clearly reported that the sensor was most selective to 3-MP compared with other chemicals (**Fig 10B**). The sensitivity of the Fe_3_O_4_.CNT NCs coated electrode sensor was performed up to two weeks for the examination of the reproducible and storage capabilities. It was recognized that the I-V responses were not significantly changes after washing of each experiment of the fabricated Fe_3_O_4_.CNT NCs electrode (**Fig 10C**).

The sensitivity remained almost equal as the initial response up to two weeks and after that the responses of the fabricated electrode become decreased gradually. A series of six successive measurements of 3-MP solution (900 nM) yielded good reproducible responses with the Fe_3_O_4_.CNT NCs electrode at different conditions. A control experiment was also performed at 3-MP concentration (900 mM) with different fabricated electrodes and a remarkable increased of current response was found for the Fe_3_O_4_.CNT NCs compared with Fe_3_O_4_ NPs (**Fig 10D**). The responses of NCs sensor were determined with respect to storage time for measurement of long term storage capacity. The storage stability measurement of the Fe_3_O_4_.CNT NCs electrode sensor was conducted under normal conditions and the sensitivity remained almost 90% as the initial responses for several days. It was clearly denoted that the fabricated sensor may be used without any significant degradation of sensitivity up to several weeks. The sensor performances using different electrochemical approach toward phenolic derivatives have been concluded [24, 49, 63–65] in Table 2.

**Table 2:** Detection of phenols using different electrochemical approach.

Electrode	Methods	Phenols	Sensitivity (μAμM^-1^cm^-2^)	LOD	LDR	Ref.
				(pM)	(mM)	
**POAS-Ag/MWCNT/GCE**	I-V	3-MP	3.829 μAmM^-1^cm^-2^	360	0.4–40.0	[24]
**NiO.CNT/GCE**	I-V	4-AP	6.33 × 10^−4^	15	-	[49]
**Graphene-polyaniline/GCE**	DPV	4-AP	1.776042	0.065 mM	0.2–20, 20–100	[63]
**RGO/P-**_**L**_**-GSH/GCE**	AM	4-AP	27.2	0.03 mM	0.4–200	[64]
**Ce**_**2**_**O**_**3**_**.CNT/GCE**	I-V	2-NP	1.6 × 10^−3^	60	100.0 pM -100.0 μM	[65]
**Fe**_**3**_**O**_**4**_**.CNT NCs/GCE**	**I-V**	**3-MP**	**9.49 × 10**^**−4**^	**1.0**	**90 pM– 90 nM**	**This work**

4-AP = 4-Aminophenol, AM = Amperometry, 3-MP = 3-Methoxyphenol, 2-NP = 2-Nitrophenol.

### Real sample analysis

On the subject of authentication of the legitimacy of I-V system, the Fe_3_O_4_.CNT NCs/GCE used to detect the 3-MP in different original samples. A standard addition method used to approximate the concentration of 3-MP in real samples that were collected from diverse sources. A set amount (~25.0 μL) of every original analyte mixed and examined in PB (10.0 mL) using fabricated Fe_3_O_4_.CNT NCs/GCE. The obtained results concerning 3-MP finding are presented in Table 3, and actually established that the anticipated Fe_3_O_4_.CNT NCs/GCE advancement is acceptable, dependable, and proper for analyzing real samples using I-V design.

**Table 3:** Measurement of 3-MP using modified Fe_3_O_4_.CNT NCs/GCE.

Real samples	Observed current (μA)				Conc. (μM)	SD
	R1	R2	R2	Average		(n = 3)
Industrial effluent	7.09	5.27	4.91	5.75	23.76	1.17
PC baby bottle	7.73	5.25	4.42	5.80	23.96	1.72
PC bottle safa	1.66	4.14	3.85	3.22	13.30	1.36
PVC food packaging bag	4.18	3.08	2.77	3.34	13.80	0.74
Red sea water	4.33	3.31	2.58	3.41	14.09	0.88
Tape water	3.54	2.66	2.31	2.83	11.71	0.63

R = Reading, SD = Standard deviation

## Conclusion

Fe_3_O_4_.CNT NCs were prepared using an easy, efficient and simple wet-chemical method in basic medium. The electrochemical characteristic of NCs was performed by UV/Vis, FT-IR, FESEM, XEDS, XPS and XRD techniques. A simple fabrication method used to fabricate Fe_3_O_4_.CNT NCs thin-film onto flat GCE electrode. The sensitive and selective of 3-MP sensor was prepared successfully based on GCE embedded with Fe_3_O_4_.CNT NCs by conducting coating binder. The electrochemical investigation of the fabricated 3-MP sensor was excellent in point of detection limit including linear-dynamic range, sensitivity and response time. The Fe_3_O_4_.CNT NCs/GCE exhibited higher sensitivity (9×10^−4^ μAmM^-1^cm^-2^) and lower detection limit (1.0 pM) by considering the signal-to-noise ratio of 3. A well-established route can be introduced from this novel approach for the development of efficient chemical sensor for biological and environmental toxin in a broad scale.

## Supporting information

S1 Fig Preparation of nanocomposites from Fe_3_O_4_ NPs and CNT. (DOCX)
