# Supplementary material for: Fabrication of 3-methoxyphenol sensor based on Fe3O4 decorated carbon nanotube nanocomposites for environmental safety: Real sample analyses
Source: PLoS One. 2017 Sep 22;12(9):e0177817. doi: 10.1371/journal.pone.0177817 (PMC5609863; doi:10.1371/journal.pone.0177817)
Supplement: S1 Fig — (DOCX) [file pone.0177817.s001.docx]

**Supporting Information**

**S1 Fig. Preparation of nanocomposites from Fe_3_O_4_ NPs and CNT:**

The wet-chemical technique is a typical solid-state synthesis method and extensively used in the preparation of undoped or doped nanostructure materials. The products can be obtained as minor grains including shorter duration of phase formation. FeSO_4_.7H_2_O, CNT, and NaOH were used as active agents in preparation of Fe_3_O_4_.CNT NCs. Following of this procedure, CNT (1.0 wt %, 0.25 μg) was added under continuous stirring with dissolved FeSO_4_.7H_2_O (100 mM, 2.78 g) in distilled water (100.0 mL) at a conical flask (250.0 mL). pH of the resultant mixture was adjusted to 10.35 by adding NaOH and then placed for continuous stirring (6 h) at 90.0 °C. After 6 h constant stirring, the flask was washed thoroughly with water and acetone consequently, and then kept for drying (24 h) in open air at room temperature (R. T). The resultant brown color products (Fe_3_O_4_.CNT NCs) were dried (28 h) in the oven at 60.0 °C, grinded into powders, and dried again (22 h) at 60.0 °C in oven in order to use for electrochemical characterizations and application as chemical sensor. The Fe_3_O_4_ NPs without CNT was also prepared using the same procedure. The probable reactions mechanism for the formation of Fe_3_O_4_.CNT NCs is as follows.

| **NaOH _(s)_ → Na^+^ _(aq)_ + OH^-^ _(aq)_** | **(i)** |
| --- | --- |
| **FeSO_4_ → Fe^2+^ _(aq)_ + SO_4_^-2^ _(aq)_** | **(ii)** |
| **2Na^+^ _(aq)_ + 2OH^-^ _(aq)_ + Fe^2+^ _(aq)_ + SO_4_^-^ _(aq)_ → Fe(OH)_2 (aq)_ + Na_2_SO_4 (aq)_** | **(iii)** |
| **Fe(OH)_2 (aq)_ → Fe_3_O_4_ _(s)_↓ + H_2_O _(aq)_** | **(iv)** |
| **Fe_3_O_4_ + CNT (dispersed) →Fe_3_O_4_.CNT _(s)_↓** | **(v)** |
